# A Bimetallic-Coated, Low Propagation Loss, Photonic Crystal Fiber Based Plasmonic Refractive Index Sensor

**DOI:** 10.3390/s19173794

**Published:** 2019-09-01

**Authors:** Mohammad Al Mahfuz, Md. Anwar Hossain, Emranul Haque, Nguyen Hoang Hai, Yoshinori Namihira, Feroz Ahmed

**Affiliations:** 1 Department of Electrical and Electronic Engineering, Independent University Bangladesh (IUB), Dhaka 1229, Bangladesh; 2 Department of Electrical and Electronic Engineering, Green University of Bangladesh, Dhaka 1207, Bangladesh; 3 School of Electronics and Telecommunication, Hanoi University of Science and Technology, Hanoi 100000, Vietnam; 4 Faculty of Engineering, University of the Ryukyus, Nishihara 151-0066, Japan

**Keywords:** photonic crystal fiber (PCF), confinement loss, wavelength sensitivity, plasmonic sensor, refractive index (RI)

## Abstract

In this paper, a low-loss, spiral lattice photonic crystal fiber (PCF)-based plasmonic biosensor is proposed for its application in detecting various biomolecules (i.e., sugar, protein, DNA, and mRNA) and biochemicals (i.e., serum and urine). Plasmonic material gold (Au) is employed externally to efficiently generate surface plasmon resonance (SPR) in the outer surface of the PCF. A thin layer of titanium oxide (TiO_2_) is also introduced, which assists in adhering the Au layer to the silica fiber. The sensing performance is investigated using a mode solver based on the finite element method (FEM). Simulation results show a maximum wavelength sensitivity of 23,000 nm/RIU for a bio-samples refractive index (RI) detection range of 1.32–1.40. This sensor also exhibits a very low confinement loss of 0.22 and 2.87 dB/cm for the analyte at 1.32 and 1.40 RI, respectively. Because of the ultra-low propagation loss, the proposed sensor can be fabricated within several centimeters, which reduces the complexity related to splicing, and so on.

## 1. Introduction

Surface plasmon resonance (SPR)-based sensing technology has added a new dimension in plasmonic science, as this phenomenon is highly capable of detecting very small refractive index (RI) changes in the surrounding medium [1,2]. The SPR sensing phenomenon can be efficiently demonstrated between the metal–dielectric interface when the frequency of the incoming light and the frequency of the free electrons mutually coincides [3]. SPR sensors are used for different sensing applications such as in diagnostics for health, environmental monitoring, and biochemical and bio-organic sample detection [4,5]. Current SPR-sensing platforms are based on prism coupling, optical fibers, and fiber gratings [6]. However, prism coupling based optical devices are bulky in their configuration and not feasible for integration and miniaturization [7]. Optical fiber based sensors offer a high resolution, a miniaturized structure, and are also capable in remote sensing applications [8]. In contrast to conventional optical fiber, photonic crystal fibers (PCFs) are highly acceptable for SPR-sensing purposes because of their unique, controllable light-guiding mechanism, excellent birefringent properties, and flexible structural design [9,10]. However, most of the reported PCF-SPR sensors have a high propagation loss and comparatively low sensitivity. Therefore, the current PCF-SPR sensing research can be tailored based on low propagation loss with high sensitivity. Owing to the analysis and improved sensitivity response, the sensor’s performance can be increased by varying the structural parameters [11] such as air hole dimensions, pitch, plasmonic metal thickness, fiber length, ring numbers, and lattice configuration (i.e., hexagonal, circular, spiral, rectangular, or octagonal).

Plasmonic material selection is another key issue for the PCF-SPR sensor. To date, various well-known plasmonic materials are used to excite SPR such as gold [12,13], silver [4], copper [2], graphene [14], and titanium nitride [15]. Recently, thin layers of oxides such as indium–tin oxide (ITO), aluminum oxide (Al_2_O_3_), and titanium oxide (TiO_2_) have also been used [16,17,18], along with plasmonic materials, to form a bimetallic layer, which assists in creating a strong SPR effect. The bimetallic layer also assists in increasing wavelength sensitivity and sensing range. Until now, various PCF structures have been proposed—internal metal-coated [19], external metal-coated [20], side-polished [21], *D* shape [22], modified *D* shape [23], open channel [24], and slotted [25]—to improve the sensing response. Rifat et al. proposed a *D*-shaped Au-TiO_2_ bimetallic coated plasmonic sensor [7], which shows the highest loss of 23.18 dB/cm, an average wavelength sensitivity of 9800 nm/RIU, and a resolution of 2.2 × 10**^−^**^6^ RIU. However, to make a *D*-shaped flat surface, excessive surface polishing is needed. Recently, Zhang et al. proposed a grooved, microstructure-coated PCF-SPR sensor [26], which showed a maximum loss of 140.3 dB/cm, an improved wavelength sensitivity of 15,933 nm/RIU, and a maximum wavelength resolution of 6.84 × 10**^−^**^6^ RIU in the measurement range from 1.40–1.43. Therefore, the internal microstructure coating makes this sensor critical during fabrication. To overcome the above limitations, an external sensing method can be utilized by placing the sensing medium as well as plasmonic metal at the outer surface of the PCF. On that perspective, until now, different types of circular PCF-SPR sensors have been developed [27,28,29]. Liu et al. proposed a single-layer birefringent sensor [27], which showed the highest leakage loss at 110 dB/cm, a wavelength sensitivity of 15,180 nm/RIU, and a sensor resolution of 5.68 × 10**^−^**^6^ RIU in the sensing range between 1.40 and 1.43. Most Recently, Lou et al. proposed a gold–graphene bimetallic sensor [28], which exhibited the highest propagation loss of 185.5 dB/cm and maximum wavelength sensitivity of 8600 nm/RIU in the sensing range from 1.33 to 1.38. All the above sensors have a hexagonal or circular lattice configuration. In addition, Hasan et al. proposed a spiral lattice dual-polarized sensor [29], which showed the highest peak loss at 22.63 dB/cm and wavelength sensitivity of 4600 nm/RIU. However, from the above literature review, it is clear that the wavelength sensitivity is moderate, and the propagation loss is very high.

In this paper, a simple, low-loss, PCF-based SPR sensor is proposed to promote the detection of analytes in the visible to near-IR wavelengths in real-time. The plasmonic metal layer and the analyte layer are effectively placed on the outer portion of the PCF surface to reduce fabrication complexity. In the cladding region, all lattice air holes are circular, and the hole dimensions are also identical, which can make this sensor more feasible for practical realization. To utilize the sensing performances, the impact of plasmonic materials with varying Au thicknesses, TiO_2_ thicknesses, and the impact of the Au-TiO_2_ bimetallic layer compared to the only Au layer are studied. Later, the air hole dimensions and pitch parameter effects are also studied, showing a corresponding normalized loss intensity. Finally, the feasibility of fabricating the proposed sensor is discussed. 

## 2. Sensor Design and Brief Theory

**Figure 1:**
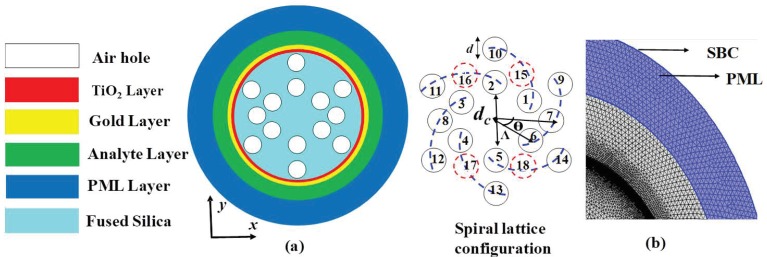
(**a**) Two-dimensional cross-section view of the proposed spiral photonic crystal fiber (PCF) sensor. (**b**) Computational meshing domain with optimized design parameters. PML, perfectly matched layer; SBC, scattering boundary condition.

A two-dimensional schematic cross-section of the proposed PCF sensor is depicted in Figure 1, where the array of air holes is well organized in a spiral configuration. This sensor consisted of three air hole rings with six arms. In the first ring, the first hole was placed at a 30° position, and the other holes, such as numbers 2, 3, 4, 5, and 6, were placed at 90°, 150°, 210°, 270°, and 330° positions, respectively. The difference of the angular position between two adjacent holes in the same arm was Θ = 30°. The central air hole (core) diameter was *d_c_* = 3 µm, which was removed to confine maximum light at that region. Two air holes in the second ring were positioned at 0° and 180° (numbers 7 and 8), and the other four air holes (numbers 15, 16, 17, and 18) have been removed from 60°, 120°, 240°, and 300° positions to excite SPR and also to stack the solid capillary for the consideration of fabrication. The regular air holes are stacked with thin wall capillary. The distance from the core to the center of the first ring of air holes was fixed at 2 µm, which is denoted as pitch (Λ). The other two pitches were considered as 1.4 Λ and 1.8 Λ for the distance from the core to the second- and third-ring air holes, respectively. The diameter of the cladding air holes was scaled at *d* = 1.3 µm. 

Fused silica glass was used as the background material of the sensor, for which the dispersion characteristics can be carried out from the following Sellmier equation [9]:

(1) $$n^{2}\left(\lambda \right)=1+\frac{B_{1}\lambda^{2}}{\lambda^{2}-C_{1}}+\frac{B_{2}\lambda^{2}}{\lambda^{2}-C_{2}}+\frac{B_{3}\lambda^{2}}{\lambda^{2}-C_{3}},$$

where *n* is the RI of the fused silica, which is dependent on wavelength λ. Here, the constants are taken from Ref. [9]. 

The complex RI of gold can be defined by the following equation from the Drude–Lorenz model [9]:

(2) $$\varepsilon_{Au}=\varepsilon_{\infty }-\frac{\omega_{D}^{2}}{\omega \left(\omega +j\gamma_{D}\right)}-\frac{\Delta \varepsilon \cdot \Omega_{L}^{2}}{\left(\omega^{2}-\Omega_{L}^{2}\right)+j\Gamma_{L}\omega },$$

where ε_Au_ is the gold permittivity. All constant values of Equation (2) are taken from Ref. [9].

A thin layer of TiO_2_ was also used between gold and silica, which assists in reducing the adhesion problem of Au and improves sensitivity. The TiO_2_ layer was also helpful for exciting the SPR with efficiently contacting the core-guided mode to the SPP mode [18]. The dielectric constant of titanium oxide is calculated by the following equation [30]:

(3) $$n_{TiO_{2}}^{2}=5.913+\frac{2.441\times 10^{7}}{\left(\lambda^{2}-0.803\times 10^{7}\right)},$$

where $n_{TiO_{2}}$ is the wavelength-dependent RI of titanium oxide, and λ is the wavelength measured in µm.

A crucially important performance parameter for the proposed PCF-SPR sensor is confinement loss, which can be evaluated by the following expression [5]:

(4) α(dB/cm)=8.686×koIm(neff)×104,

where the imaginary effective mode index is denoted as Im (*n_eff_*), the wave number is *k*_0_ = 2*π*/*λ*, and the operating wavelength is *λ*.

Analyte sensing occurs with small variations of the wavelength of the bio-targets in the surrounding environment. Therefore, wavelength sensitivity is also an important performance parameter of the PCF-based SPR sensor, which can be calculated by the following expression [18]:

(5) $$S_{\lambda }(nm/RIU)=\;\Delta \lambda_{\mathrm{peak}}/\Delta n_{a},$$

where Δ*n_a_* denotes the refractive index difference of the two adjacent analytes, and Δλ_peak_ means the variations of the two nearby resonance peaks.

Another parameter for calculating the sensing performance is sensor resolution, which can be determined from the following equation [18]:

(6) $$R(RIU)=\Delta n_{a}\times \Delta \lambda_{\min}/\Delta \lambda_{peak},$$

where the two nearby dielectric RI variations are considered as Δ*n_a_* = 0.01, and the minimum wavelength peak resolution is considered as Δλ_min_ = 0.1 nm. Here, the noises originated by the external perturbation, and also the instrumental noise, are effectively ignored during wavelength resolution calculations.

The sensing performance, as well as the numerical analysis of the proposed sensor, was performed by using the commercially available software *COMSOL Multiphysics 5.4*. Figure 1b represents the computationally extreme and fine meshing domain with optimized design parameters of *d* = 0.65Λ, *d_c_* = 3 µm, Λ = 2 µm, *T_t_* = 10 nm, and *t_g_* = 40 nm. The total mesh consisted of a total of 97,610 triangular elements, where the edge and vertex elements were 5680 and 76, respectively. The total mesh area was 181.4 µm^2^. Scattering boundary conditions (SBCs) and perfectly matched layers (PMLs) were applied to obtain better computational accuracy during the simulation. The PML layer effectively absorbs the scattering photon and prevents unintended reflections. The dielectric or sensing layer was introduced between the PML layer and the gold layer.

**Figure 2:**
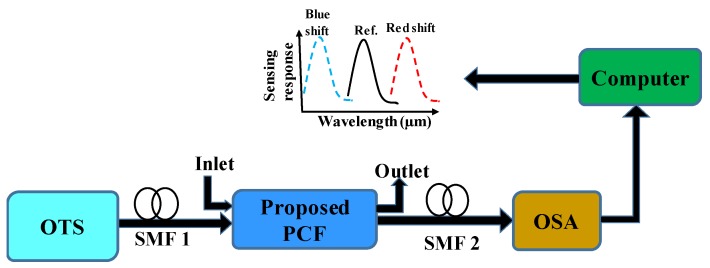
Schematic representation of the experimental setup of the proposed sensing platform. OSA, optical spectrum analyzer; OTS, optical tunable source; SMF, single-mode fiber.

Though the proposed work is conducted on a fully theoretical basis, the schematic experimental setup of the sensing system for our sensor is described in Figure 2. This setup included the optical tunable source (OTS), polarization controller, and optical spectrum analyzer (OSA). These components are connected with a single-mode fiber (SMF). The analyte or sensing layer was positioned at the outer portion of the PCF, and the inlet (analyte) and outlet (analyte) can be controlled via a pump. When mutual interaction between the analyte (sensed by the RI) and legend takes place, then a small blue shift (shifted to the shorter wavelength) or red shift (shifted to the longer wavelength) of the loss peak is observed, which can be easily monitored via OSA. The principal of the red or blue shift can be described from the following equation [30]:

(7) $$d_{p}=\frac{1}{k\beta }=\frac{\lambda }{2\pi \beta },$$

where *d_p_* means the penetration depth generated by the evanescent wave, and *β* and *k* are the decay constant and wave number. From the equation, it can be concluded that the incident wavelength is proportional to *d_p_*. Hence, for a longer wavelength *d_p_* is high, and for a shorter wavelength *d_p_* is low. Strong coupling is observed between the core-guided mode and plasmonic mode for longer wavelengths, resulting in comparatively more damping of the evanescent wave. Therefore, the red shift phenomenon occurs. On the contrary, for the reverse reason, blue shift can happen. By changing the RIs of the environmental bio-targets surrounding the PCF, the unknown analytes can be detected by analyzing the output loss spectrum in the computer.

## 3. Numerical Performance Analysis and Brief Discussion

### 3.1. Dispersion Relation and Optical Field Distribution with Coupling Strength

**Figure 3:**
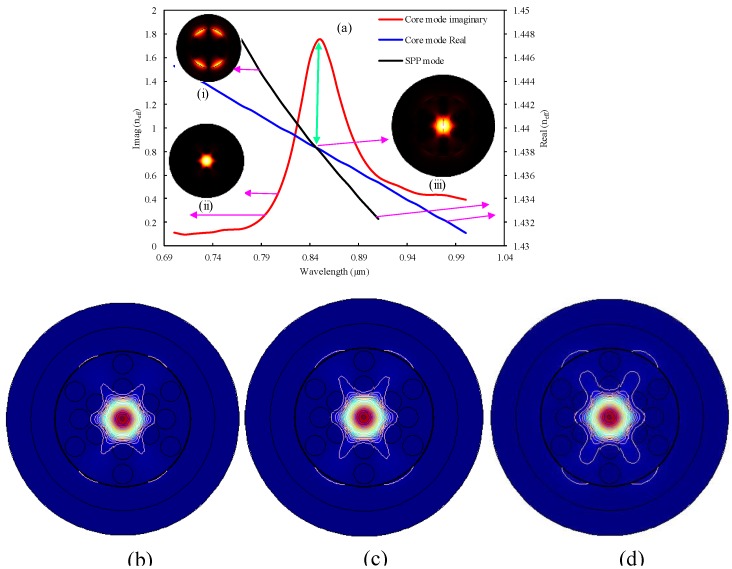
Complex refractive index (RI) of the sensor and optical field distribution (**a**) (inset (**i**) SPP mode, (**ii**) core mode, and (**iii**) resonance condition) for analyte RI of 1.39, (**b–d**) representation of coupling strength for different analyte RIs from 1.38–1.40 with using the optimized sensor parameters.

Figure 3a shows the dispersion relation as well as optical field distribution for the resonance condition. The inset shows the (i) SPP mode, (ii) core mode, and (iii) coupling of the SPP and core-guided mode. Mathematically, resonance occurs when the real part of the effective mode index (*n*_eff_) of the core-guided mode and the SPP mode coincide [5]. At the resonance wavelength, a sharp loss peak was observed, and unknown samples could be effectively determined by shifting this peak to a longer or shorter wavelength for different analyte refractive indexes. Imaginary (confinement loss) and real effective mode indexes for the core-guided mode are shown in Figure 3a by red and blue lines. Additionally, the black line shows the SPP mode for the interaction of the evanescent wave on the gold–dielectric surface. The real part of the SPP and core-guided modes intersected at a wavelength of 0.85 µm, and a peak was observed at the point of intersection, which is the phase-matching condition for the analyte RI of 1.39. At this point, maximum energy transfers from the core-guided mode to the plasmonic mode.

### 3.2. Influence of Analyte RI (n_a_) Variations on Sensing Characteristics 

**Figure 4:**
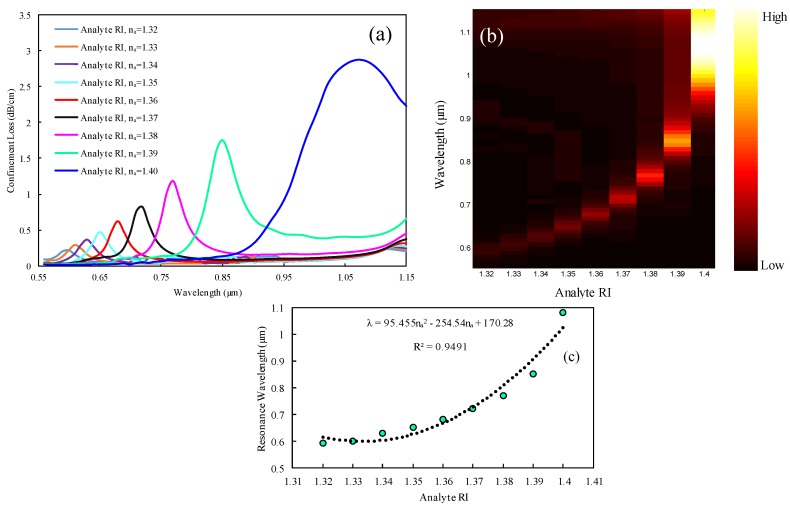
(**a**) Confinement loss spectra, (**b**) normalized resonance intensity, and (**c**) polynomial fitting characteristics for analyte RIs variation from 1.32 to 1.40.

The analyte’s RI variation has a significant impact on PCF-SPR-sensing characteristics. The SPR-sensing mechanism with PCF is susceptible to the surrounding environment. In contrast with the other sensing technique, the proposed mechanism showed a comparatively larger resonance peak shift when small RI variations in the targeted dielectric occurred. For this analysis, the *y*-polarized mode was considered as it exhibited a comparatively larger evanescent field, resulting in the propagation of maximum free-surface electrons, compared to the *x*-polarization mode. From Figure 4a, it can be seen that the resonance peak of the confinement loss curve became sharper and gradually broader (redshifted) to the longer wavelength, with a varying RI of analyte (*n_a_*) from 1.32 to 1.40. This phenomenon can be described as follows: with the increase of analyte RIs, the effective mode index of the sensing medium was reduced significantly, and the RI contrasts also decreased between the SPP mode and the core-guided mode. Owing to the small RI contrast, maximum light penetrated through the cladding region instead of light confinement through the core region, resulting in a comparatively higher amount of light that vibrated through the metal surface and coupled with the dielectric (this phenomenon is demonstrated well in the contour plot in Figure 3b–d). That is the reason a redshifted peak was obtained with high loss. Using Equation (4), a resonance loss peak of 0.22 dB/cm was obtained at 0.6 µm for *n_a_* of 1.32, and a resonance peak of 0.28 dB/cm was observed at 0.61 µm for *n_a_* of 1.33. Here, the wavelength variation was 0.01 µm. Therefore, the wavelength sensitivity obtained was about 1000 nm/RIU by using Equation (5). Similarly, the resonance mode loss peaks gradually increased from 1.34 to 1.40, respectively, and the highest resonance mode loss peak for this sensor was obtained at about 2.87 dB/cm at 1.08 µm for *n_a_* of 1.40. The resonance mode loss peak shifted from 0.85 to 1.08 µm for an analyte RI from 1.39 to 1.40. As a result, the highest wavelength sensitivity was calculated at about 23,000 nm/RIU. Using Equation 6, we can calculate the corresponding wavelength resolution by considering the minimum 1% sensing capability. The proposed sensor showed a minimum wavelength resolution of 1 × 10^−4^ RIU for an *n_a_* variation from 1.32 to 1.33 and a maximum wavelength resolution of 4.34 × 10^−6^ RIU for an *n_a_* variation from 1.39 to 1.40. Details of the simulation results are organized in Table 1. Figure 4b depicts the normalized mode loss intensity for different *n_a_* variations from 1.32 to 1.40. From the figure, it is evident that the minimum intensity was obtained for *n_a_* of 1.32, and the maximum intensity was obtained for *n_a_* of 1.4. This is the reason behind the fact that the mutual interaction between plasmonic and core-guided modes were strengthened for higher analyte RI values and, on the contrary, remained weak for lower analyte RI values. Therefore, a strong interaction results in a high intensity, and a comparatively weak interaction results in a low intensity. The adjacent color bar shows a black portion as low intensity and a yellow-white portion for high intensity.

Investigation of polynomial fitting of the proposed sensor is also crucial for better optimization. The average sensitivity can be measured by this polynomial fitting curve, which is depicted in Figure 4c. In the figure, the dotted line represents the polynomial fitting, and the marker means the resonance wavelength for the respective analyte RIs. The relationship between analyte RI and resonance wavelength can be measured by the R^2^ value in the measurement range from 1.32 to 1.40. The proposed spiral PCF sensor showed that R^2^ = 0.9491. The corresponding polynomial regression equation was *λ* = 95.455*n_a_*^2^ – 254.54*n_a_* + 170.28, where *n_a_* represents the analyte RI and *λ* represents the resonant wavelength.

### 3.3. Impact of Plasmonic Material Thicknesses on the Sensing Performance

**Figure 5:**
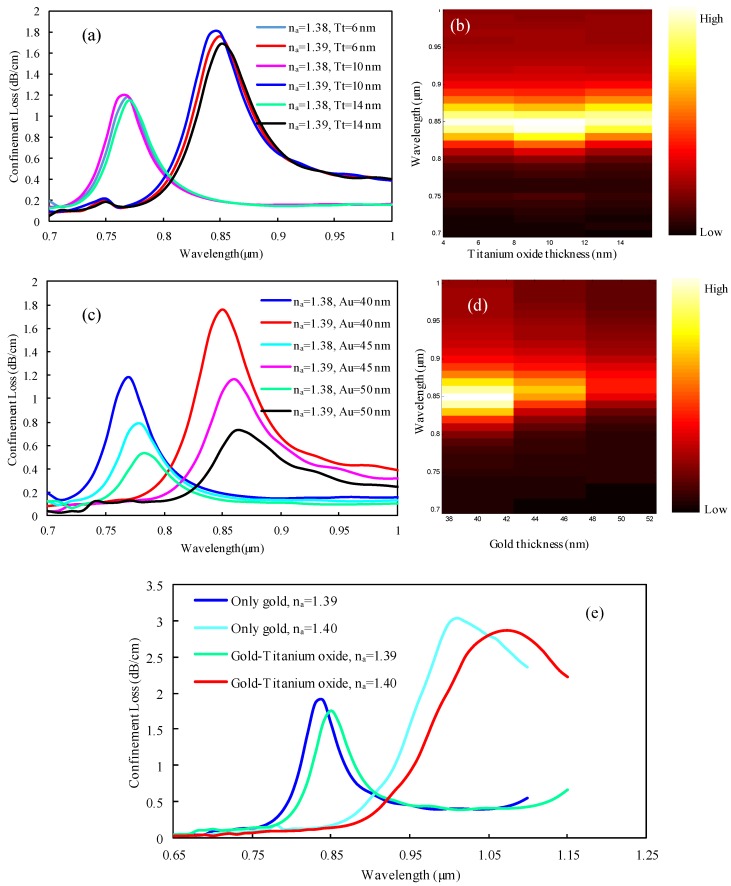
Observation of loss spectrum for (**a**) TiO_2_ thickness variations and (**c**) Au layer thickness variations; (**b**) and (**d**) represent the normalized loss intensity for TiO_2_ and Au thickness variations for *n_a_* of 1.39; (**e**) plasmonic material effects of the Au-TiO_2_ bimetallic layer and Au alone using optimized sensor parameters.

The impact of TiO_2_ and Au layer thicknesses on sensing performance is illustrated in Figure 5. From Figure 5a it is evident that, when the thickness of the TiO_2_ layer was raised from 6 to 14 nm, a negligible variation of mode loss peak was observed. Loss resonance peaks of 1.1859, 1.1814, and 1.143 dB/cm, respectively, were obtained at 0.77 µm wavelength for n_a_ of 1.38, and the highest loss peaks of 1.7954, 1.7546, and 1.6792 dB/cm were obtained at 0.85 µm wavelength, respectively, for *n_a_* of 1.39. It is notable that, here, the wavelength sensitivities were identical at 8000 nm/RIU for all considered TiO_2_ layer thickness variations from 6 to 14 nm. Also, from Figure 5b it is shown that around 0.85 µm resonance wavelength, the normalized mode loss intensity was slightly higher for 10 nm thickness for *n_a_* of 1.39. Hence, our optimized TiO_2_ layer thickness was (*T_t_*) = 10 nm. 

Au plasmonic material has a notable impact on PCF-SPR sensor performance. The impact of variations in the Au layer thickness is demonstrated in Figure 5c–d. From Figure 5c is evident that, with an increment of the gold layer thickness from 40 to 50 nm, the loss resonance peak slightly redshifted, and the loss peak showed a downward tendency. The reason behind this phenomenon can be explained as follows: because of the damping characteristics of gold, the loss resonance peak decreases for a thicker layer of gold. In contrast, the thinner layer of gold increases the loss depth. Penetration depth also has significant impacts on mode loss and resonance intensity of loss. The frequency of the incoming photon is proportional to the penetration depth [30]. Therefore, a thicker gold layer needs a higher wavelength for light penetration. Hence, the loss peak redshifts with an increasing gold layer thickness.

With an increment of gold layer thickness from 40 to 45 and 50 nm, loss peaks were reduced to 1.0.787 and 1.1611 for n_a_ of 1.38 and 0.524 and 0.726 dB/cm for n_a_ of 1.39. Here, the resonance wavelength redshifted to 1000 nm for 45 and 50 nm thicknesses, respectively. However, the wavelength sensitivity was at 8000 nm/RIU for all considered thicknesses. Additionally, from Figure 5d it can be predicted that the strongest propagation mode loss intensity was obtained for the 40 nm thickness of gold, and the lowest intensity was seen for the 50 nm thickness of gold. Therefore, we carefully chose the optimized gold layer thickness of *t_g_* = 40 nm to attain a higher sensitivity as well as better light interaction from the core-guided mode to the SPP mode.

Because of its unique damping properties and chemical stability in the environment, Au is considered as a key plasmonic material. A thin TiO_2_ layer can be used to assist the adhesion between Au and silica, which also improves the sensing performance. For selecting plasmonic material for our sensor, we compared the sensing performances by using only the Au layer and by using the Au-TiO_2_ bimetallic layer. Figure 5e shows that when the Au-TiO_2_ materials were used, then the resonance peak of mode loss was observed about 1.7546 dB/cm at 0.85 µm and 2.87033 dB/cm at 1.08 µm for *n_a_* values of 1.39 and 1.40, respectively. Moreover, the corresponding wavelength sensitivity obtained was about 23,000 nm/RIU. However, when only the Au material was used, then the resonance peak of mode loss was reduced and was about 1.8943 dB/cm at 0.84 µm and 3.07 dB/cm at 1.01 µm for analyte RIs of 1.39 and 1.40, respectively. Therefore, the corresponding wavelength sensitivity obtained was about 17,000 nm/RIU. From Figure 5e it is seen that, when only the Au material was used, then the mode loss increased, and the corresponding wavelength sensitivity was dramatically reduced. The wavelength sensitivity was highest, and propagation loss was lowest, for the Au-TiO_2_ bimetallic layer rather than only the Au layer. Hence, we used the Au-TiO_2_ bimetallic layer instead of using only the Au layer in our proposed sensor.

### 3.4. Influence of Λ and d Variations on the Sensing Performance

**Figure 6:**
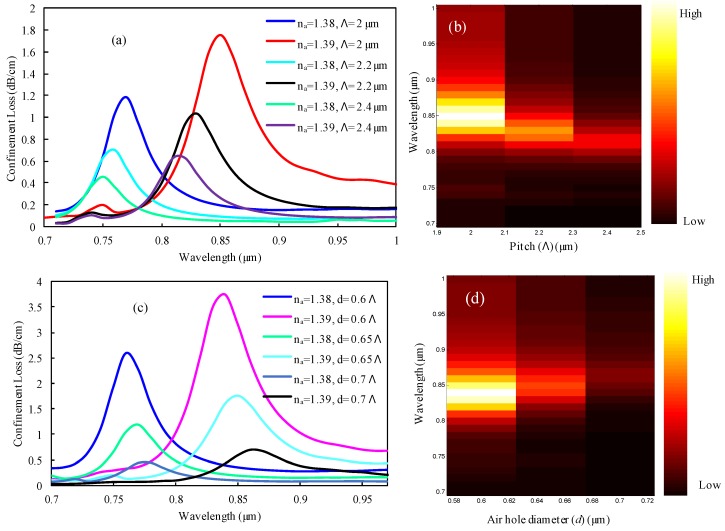
Representation of loss spectrum for (**a**) pitch (Λ) variation from 2 to 2.4 µm, (**c**) air hole diameter, and (**d**) variation from 0.6 Λ to 0.7 Λ for *n_a_* values of 1.38 and 1.39; (**b**) and (**d**) represent the loss intensity for Λ and *d* variation for *n_a_* of 1.39.

The analyte sensing characteristics of the PCF-SPR sensor are also dependent on structural design parameters such as pitch (Λ) and air hole diameter (*d*). Owing to the variation of Λ and *d*, the detection properties change simultaneously. When Λ is increased, then the mutual interaction between the core-guided mode and the SPP mode is reduced. Therefore, the blueshifted loss peak observed and the resonance intensity also decreased monotonically. As illustrated in Figure 6a the mode loss peak decreased with a negligible blue shift in order to increase the pitch dimension from 2 to 2.4 µm. The highest loss peaks of 1.1814 and 1.7546 dB/cm were observed at 0.77 and 0.85 µm for *n_a_* values of 1.38 and 1.39, respectively, with a pitch parameter of 2 µm. In addition, the highest loss peak was obtained at about 0.69 and 0.45 dB/cm for *n_a_* values of 1.38 and 1.39, respectively, with a pitch dimension of 2.2 µm. Moreover, loss peak was observed at about 1.02 and 0.62 dB/cm for *n_a_* values of 1.38 and 1.39, respectively, with a pitch dimension of 2.4 µm. In addition, the wavelength sensitivity also decreased from 8000 to 7000 nm/RIU for Λ values of 2 to 2.4 µm, respectively. Resonance intensity variations are shown in Figure 6b, where the highest intensity was observed for Λ = 2 µm. Hence, we considered Λ = 2 µm as the optimized pitch parameter for this proposed structure. Furthermore, a similar phenomenon was observed for the increment of diameter *d* as the Λ variation, which is depicted in Figure 6c–d. Because of the moderate loss and overall better sensing performance, the lattice air hole diameter was carefully optimized to *d* = 0.65 Λ for the proposed design. 

Feasibility of fabrication is another key issue for the spiral lattice PCF-SPR sensor. The equiangular configuration of the spiral design of PCF can be fabricated using a stack-and-draw technique, which is also discussed in the following Refs. [29,31]. A thin layer of plasmonic metal Au and TiO_2_ can be deposited with the commonly used chemical vapor deposition (CVD) method [32], wheel polishing method [18], and atomic layer deposition (ALD) [29]. Table 2 shows performance comparisons with respect to the previously implemented sensor. From the table, it is observable that both the measurement range and wavelength sensitivity of the proposed sensor were comparable to that of the reported sensors.

## 4. Conclusions

A highly sensitive, low-loss PCF-SPR sensor has been proposed for an analyte sensing range between 1.32 and 1.40 RI. The sensing performances are realized by using the finite element method (FEM). The simulation results show that a maximum wavelength sensitivity of 23,000 nm/RIU, a very low propagation loss of 2.87 dB/cm, and a sensor resolution of 4.34 × 10^−6^ RIU were obtained. Because of the ultra-low loss and high wavelength sensitivity, the proposed sensor can be a promising candidate for application in detecting various bio-samples in the lab-on-a-fiber platform.

## Figures and Tables

**Table 1:** Performance investigation for the analyte RI detection range from 1.32 to 1.40.

Analyte RI	Resonance Mode Loss (dB/cm)	Resonance Wavelength (µm)	Wavelength Peak Shift (nm)	Wavelength Sensitivity (nm/RIU)	Wavelength Resolution (RIU)
1.32	0.2219	0.6	10	1000	1 × 10^−4^
1.33	0.2853	0.61	20	2000	5 × 10^−5^
1.34	0.36246	0.63	20	2000	5 × 10^−5^
1.35	0.46932	0.65	30	3000	3.33 × 10^−5^
1.36	0.62095	0.68	40	4000	2.5 × 10^−5^
1.37	0.81593	0.72	50	5000	2 × 10^−5^
1.38	1.1814	0.77	80	8000	1.25 × 10^−5^
1.39	1.7546	0.85	230	23,000	4.34 × 10^−6^
1.40	2.87033	1.08	-----	----------	--------

**Table 2:** Performance comparison with the previously reported PCF-SPR sensor.

References	Sensing Range	Maximum Peak Loss (dB/cm)	Wavelength Sensitivity (nm/RIU)	Wavelength Resolution (RIU)
Ref. [5] Au coated	1.33–1.40	180	12,000	8.33 × 10^−6^
Ref. [7] Au-TiO_2_ Coated	1.33–1.43	23.18	9800	2.2 × 10^−6^
Ref. [11] Au Coated	1.33–1.40	65	9000	1.1 × 10^−5^
Ref. [18] Au-TiO_2_ Coated	1.33–1.38	80	25,000	4 × 10^−6^
Ref. [24] Au Coated	1.33–1.39	375.85	5000	2 × 10^−5^
Ref. [26] Au Coated	1.4–1.43	140.3	15,933	3.5 × 10^−8^
Ref. [27] Au Coated	1.40–1.43	110	15,180	5.68 × 10^−6^
Ref. [28] Au-graphene Coated	1.33–1.38	185.5	8600	-------
[proposed] Au-TiO_2_ Coated	1.32–1.40	2.87	23,000	4.34 × 10^−6^
